# Morbidity associated with systemic corticosteroid preparation for coronary artery bypass grafting in patients with chronic obstructive pulmonary disease: a case control study

**DOI:** 10.1186/1749-8090-2-25

**Published:** 2007-06-04

**Authors:** Daniele Starobin, Mordechai Rehuven Kramer, Moshe Garty, David Shitirt

**Affiliations:** 1 Pulmonary Institute, Rabin Medical Center, Beilinson Campus, Petach Tikva and Sackler Faculty of Medicine, Tel Aviv University, Tel Aviv, Israel; 2 Recanati Center for Internal Medicine and Research, Rabin Medical Center, Beilinson Campus, Petach Tikva and Sackler Faculty of Medicine, Tel Aviv University, Tel Aviv, Israel

## Abstract

**Background:**

Coronary artery bypass grafting (CABG) is associated with high morbidity in patients with chronic obstructive pulmonary disease (COPD).

We examine the effect of preoperative systemic corticosteroids on morbidity in this setting.

**Methods:**

Ninety candidates for elective CABG participated in a prospective, open randomized trial, including 30 patients with COPD who received a single injection of a long-acting corticosteroid, 30 with COPD who received placebo, and 30 without COPD who served as controls. Primary end-points were postoperative pulmonary and nonpulmonary complications. Secondary end-points were length of hospital stay (LOS), ICU stay of less than 24 hours and more than 48 hours, duration of mechanical ventilation, and time to walking and sitting.

**Results:**

The rate of pulmonary complications was similar in the two COPD groups and in the COPD patients and controls. The placebo group had more major nonpulmonary complications than the treatment group, but the difference was not statistically significant (26% vs. 17%, *P *= NS). The non-COPD control group had significantly fewer nonpulmonary complications than the COPD patients (treatment+placebo) (33% vs 70%, *P *= 0.014) and a similar rate of pulmonary complications. There was a statistically significant difference between the treated and placebo COPD groups in ICU stay less than 24 hours (*P *≤ 0.001) and more than 48 hours (P = 0.03) and hospital stay (*P *= 0.013). On stepwise analysis, only age and number of coronary grafts were predictors of pulmonary complications.

**Conclusion:**

The use of preoperative systemic corticosteroids in patients with COPD undergoing CABG may shorten ICU and hospital stay.

## 1. Background

Thoracic surgery is associated with a 10% to 40% rate of pulmonary complications [1]. The procedure-related risk factors include high American Society of Anesthesiologists (ASA) class (46% complication rate for class IV vs. 10% for class II) and long duration of surgery (73% for surgery of > 4 hours vs. 38% for < 4 hours) [2]; the patient-related risk factors are chronic obstructive pulmonary disease (COPD), asthma, smoking, poor general health status, age above 70 years, and obesity [1]. Studies have reported mortality rates ranging from 1% to 50% in patients with COPD and asthma undergoing coronary artery bypass grafting (CABG) or other major surgery [2-7]. Patient cessation of cigarette smoking, introduction of vigorous lung-expansion maneuvers, administration of antibiotics if respiratory infection is present, and treatment of airway obstruction have all been suggested as preventive measures in these patient groups [8]. Moreover, several researchers have shown that prophylactic treatment with systemic corticosteroids is safe in patients with severe asthma and COPD [2,4,9] and may help them to tolerate surgery better with fewer complications [3,9]. This is true even for severe corticosteroid-dependent asthmatics [9]. However, the efficacy by route of administration (systemic versus inhaled corticosteroids) has not yet been investigated [1-4,8-12]

The aim of the present study was to compare the benefit of preoperative parenteral administration of long-acting corticosteroids (CS) with placebo in patients with COPD undergoing elective CABG.

## 2. Patients and methods

### Patients

The study population consisted of 90 candidates for elective CABG, 60 with COPD and 30 with other disorders, who were treated at the Pulmonary Institute and Department of Thoracic and Cardiovascular Surgery of a tertiary- care hospital between February 1, 2004 and January 31, 2005. The diagnosis of COPD was based on the American Thoracic Society criteria; specifically, forced expiratory volume in 1 second (FEV_1_) less than 70% and FEV_1_/forced vital capacity (FVC) lower than 70% of predicted values [10]. Patients were also classified according to the FEV1 based on the ATS statement [10]. Both smokers and nonsmokers were included. Patients who had an upper respiratory tract infection or an exacerbation of COPD, patients who were receiving mandatory oral (more than 5 mg) or parenteral corticosteroid treatment for one month before the study, patients who required emergency CABG and patients with asthma were excluded from the study. All the study population used bronchodilators perioperatively.

The Human Ethics Committee of our hospital approved the study, and all patients signed an informed consent form prior to entry.

### Procedure

Two to three weeks prior to surgery, the 60 patients with COPD were randomly assigned to receive either a single intramuscular injection of slow-release betamethasone dipropionate 5 mg and rapid-release betamethasone sodium phosphate 2 mg (Diprospan^®^), which is equivalent to 60 mg of prednisone with a duration of action of up to 2–6 weeks (n = 30), or placebo (NaCl 0.9% injection) (n = 30). The remaining 30 patients without COPD served as a control group.

All other medications remained unchanged during the study period.

### Assessment

The patients were stratified by grade of left ventricular dysfunction and severity of obstructive lung disease, as determined by the ASA classification [21]. Cardiac and left ventricular assessment was performed by echocardiography with the SONOS-2000 device (Andover, MA, USA). Pulmonary function was assessed with the MedGraphics-Pulmonary Function System 1070 Series 2 (St.- Paul, MN, USA) at the time of randomization, and with a portable spirometer (Vitalograph, London, England) on the day before surgery and the day of discharge.

#### 2.4. Study end-points

Primary end-points of the study were postoperative pulmonary complications (atelectasis, pneumonia, pneumothorax, bronchospasm, retained secretion, sustained pleural effusion and respiratory failure) and nonpulmonary complications (arrhythmias, renal failure, heart failure, infections, bleeding, repeated surgery).

Secondary end-points were length of stay (LOS) in the intensive care unit (ICU), ICU stay less than 24 hours, ICU stay more than 48 hours, duration of mechanical ventilation and chest tube use, LOS in hospital, and intensity of rehabilitation (time to sitting and walking).

Patients were followed for 2 weeks after discharge by either a clinic visit or a phone call.

### Statistical analysis

Pearson correlation coefficients (r) and the significance for them (p) were calculated between the variables. To analyze statistically significant differences in categorical variables between the study groups, chi- square test or Fisher's exact test was used, as appropriate. To analyze statistically significant differences in mean continuous parameters between the study groups, analysis of variance with Duncan's multiple comparison option for pairwise comparisons was performed. To predict the number of pulmonary or nonpulmonary complications, a series of stepwise linear regression models was fitted to the data. Odds ratios were calculated from the estimates of the model. *P *values less than or equal to 0.05 were considered statistically significant.

## 3. Results

### Baseline demographic data

All 90 patients completed the 2-week postoperative follow-up.

As shown in Table 1, there were no differences among the treatment, placebo, and control groups patients in any of the demographic characteristics. Stratification parameters, namely, FEV_1 _and left ventricular ejection fraction, and stratification class divisions were similar in the treatment and placebo COPD groups (Tables 1, 2). FEV_1 _was significantly lower in the patients with COPD than in the controls (*P *= 0.001).

**Table 1:** Baseline patient characteristics

	Diprospan group (n = 30)	Placebo group (n = 30)	Control group (n = 30)	*P *value*
Male/Female, n	26/4	24/6	27/3	NS
Age, yr (mean ± SD)	67.0 ± 8.98	67.7 ± 9.2	66.4 ± 9.9	NS
Smokers/nonsmokers, n	24/6	26/4	20/10	NS
FEV_1_,^, ^% (mean ± SD)	60.6 ± 9.5	62.6 ± 8.0	88.6 ± 7.1	0.001
LVEF, % (mean ± SD)	46.1 ± 12.4	46.1 ± 11.8	47.8 ± 10.3	NS
ASA class, n				
2	0	0	2	
3	16	21	23	
4	14	9	5	0.04

*Between COPD groups (treatment+placebo) and controls.

**Table 2:** Distribution of patients with COPD by lung function.

	LV Dysfunction			
	
FEV_1_	Mild LV dysfunction (LVEF>35%)		Moderate to severe LV dysfunction (LVEF<34%)	
		
	Diprospan group	Placebo group	Diprospan group	Placebo group
50–70%	22	23	4	4
35–49%	2	2	1	1
< 34%	1	0	0	0

The classification is according to the ATS guidelines [10].

FEV1 = Forced expiratory volume at one second

LV = Left ventricular

LVEF = Left ventricular ejection fraction

ASA class was lower in the treatment group than in the placebo group, and significantly higher in the COPD groups (treatment and placebo) than in the control group (*P *= 0.04).

### Changes in lung function

Lung function (as defined by FEV_1_) was similar in the treatment and placebo groups at all three evaluation time points: randomization, prior to surgery, and after surgery. No significant change in mean FEV_1 _from the time of randomization to prior to surgery was noted in any of the groups. However, after CABG, there was a significant decrease in FEV_1 _in both COPD groups (*P *= 0.001). Mean FEV_1 _in the treatment group was 60.7% at the time of randomization, 61.7% prior to surgery, and 55.3% after CABG; corresponding rates in the placebo group were 62.6%, 64.3% and 54.7%.

### Operative results

Surgery parameters, including length of operation, bypass time and number of grafts, were similar in all three groups (Table 3).

**Table 3:** Operative data

	Diprospan group	Placebo group	Control group	*P *value*
Duration of surgery, hr (mean ± SD)	5.2 ± 0.97	5.1 ± 1.1	4.75 ± 0.8	NS
Duration of bypass, hr (mean ± SD)	1.7 ± 0.5	1.8 ± 0.7	1.6 ± 0.4	NS
No. of grafts (mean ± SD)	3.1 ± 0.8	3.1 ± 0.9	2.9 ± 0.8	NS
No. of LIMA bypasses	24	23	28	NS

LIMA- left internal mammary artery

*Between diprospan and placebo groups

### Primary end-points

The rates of pulmonary complications were similar in the two COPD groups (Tables 4, 5). However, there was significant difference in the rate of pulmonary complications between the COPD groups (treatment+placebo) and the control group. Although more major nonpulmonary complications were observed in the placebo group than in the treatment group, the difference did not reach statistical significance (*P *= 0.8). The patients with COPD (treatment+placebo) had more nonpulmonary complications than the patients without COPD (*P *= 0.014). Specifically, the patients with COPD had a higher rate of atrial tachyarrhythmias (36% treatment group, 26% placebo group) than the controls, and a higher rate of wound infections (20% and 23%, respectively), with or without the need for plastic reconstructive surgery (Table 5).

**Table 4:** Operative and postoperative course

	Diprospan group (n = 30)	Placebo group (n = 30)	Control group (n = 30)	*P *value*
Pulmonary complications, (%) pts.	20	20	10	NS
Nonpulmonary complications, (%) pts.	70	76	33	NS
LOS-ICU, hr (median)	93	132	34	NS
ICU<24 hours, n	19	1	3	<0.001
ICU>48 hours, n	2	8	10	0.03
Mechanical ventilation, hours (median)	55	80	32	NS
LOS-hospital, days (median)	11	13	8	0.013
Chest tube time, days (median)	3	2	2	NS
Time to sitting, days (median)	2	2	2	NS
Time to walking, days (median)	3	3	3	NS

ICU = Intensive Care Unit; LOS = length of stay

*Between Diprospan and placebo groups.

**Table 5:** Postoperative complications

Complications	Diprospan group	Placebo group	Control group	*P *value*	*P *value*?
*Pulmonary*					
-Bronchospasm	2	3	1		
-Pneumonia	1	1	1		
-Atelectasis	1	0	0		
-Pleural effusion	1	0	2		
-Pneumothorax	0	1	0		
-Respiratory failure	1	1	1		
*Total pulmonary*	6(20%)	6 (20%)	5 (10%)	0.56	0.02
*Nonpulmonary*					
Major					
- Wound infection	2	4	0		
- Acute renal failure	1	0	0		
- Stroke	1	2	0		
- Sepsis	0	1	0		
- Re-surgery	1	1	2		
*Total major*	5 (17%)	8 (26%)	2 (7%)	0.8	0.03
Minor					
- Atrial	11	8	1		
tachyarrhythmias -					
Superficial wound	4	3	4		
infection					
- Transient oliguria	1	2	3		
- Urinary tract infection	0	1	0		
- Acute gout	0	1	0		
*Total minor*	16 (53%)	15 (50%)	8 (27%)	0.1	0.01
*Total nonpulmonary*	21 (70%)	23 (76%)	10 (33%)	0.79	0.01

* Between Diprospan and placebo groups.

** Between Control and COPD groups.

### Secondary end-points

There was a statistically significant difference in ICU stay less than 24 hours (*P *< 0.001) or more than 48 hours (*P *= 0.03) between the treatment and placebo groups (Table 4). The treatment group also had a shorter LOS in hospital than the placebo group (*P *= 0.013). The duration of mechanical ventilation was longer in the placebo than the treatment group, but this difference did not reach statistical significance. There were no between-group differences in chest tube time or postoperative rehabilitation (sitting and walking) (Table 4).

### Correlation analysis

The nonpulmonary complications and length of ICU stay correlated significantly with age (*P *= 0.04), but not with left ventricular dysfunction grade, length of surgery, or bypass time. The duration of mechanical ventilation correlated significantly with FEV_1 _(*P *= 0.009) and no significantly with age, left ventricular function, length of surgery, and bypass time.

Correlation analysis showed that the risks of occurrence of both pulmonary and nonpulmonary complications increased with age, lower FEV_1_, longer ICU stay, longer mechanical ventilation, and prolonged hospitalization.

On stepwise regression analysis, age and number of coronary grafts were the most significant predictors of pulmonary complications. Specifically, pulmonary complications increased by 0.6% for each year of age and more than twofold for every additional new graft.

## 4. Discussion

The results of our study are consistent with previous reports showing that in patients with COPD, the risk of postoperative pulmonary complications rises with older age [1], lower FEV_1 _[2], and more complex coronary grafting [1]. ASA class and duration of operation are also important [13,14]. In patients who are older than 70 years or who have peripheral vascular disease, insulin-dependent diabetes mellitus, congestive heart failure, anemia, or COPD, the postoperative mortality ranges from 11% to 19% [6]. In patients with COPD, the highest pulmonary complication rates occur after CABG and major abdominal procedures (50%–60%) [5,15].

The most significant post-CABG complications have been found to be heart failure with shock, arrhythmias, pulmonary infection and atelectasis, stroke, renal failure, and surgical wound infection [6,16]. Accordingly, in the present study, the rates of nonpulmonary complications, particularly atrial tachyarrhythmias and wound infections, were quite high in the patients with COPD. One possible explanation is the poor general health status of this patient population. In our series, 23 of the 60 patients were in ASA class 4.

Sauerland et al. [17] conducted a risk benefit analysis using a meta-analysis, to compare complication rates and clinical advantages associated with the use of a single preoperative high dose of steroid in surgical patients. They found that perioperative single-shot administration of high dose methylprednisolone is not associated with a significant increase in the incidence of adverse effects. In patients with multiple fractures, limited evidence suggests promising benefits of glucocorticoids on pulmonary complications.

Michalopoulos et al. assessed the impact of history of mild-moderate COPD on outcome in patients undergoing elective CABG surgery. [18] They found that the patients with COPD have similar morbidy and mortality rates comparable to those of control.

Regarding the benefit of preoperative steroid administration in preventing postoperative complications in patients with COPD – for which previous data are lacking – we failed to find any difference in the prevalence of pulmonary complications between the patients given Diprospan and the placebo group. The treated group had fewer nonpulmonary complications than the placebo group, but the difference did not reach statistical significance. Furthermore, the patients with COPD (either pretreated or not) had a significantly higher rate of nonpulmonary complications than the control patients without COPD who underwent elective CABG. The latter findings are consistent with earlier studies [1,2,6,10,12] At the same time, we found that the patients receiving steroids had both a significantly shorter ICU stay and significantly shorter hospital stay than the patients with CPD who received placebo.

What is the possible explanation to our finding? The systemic effects of the preoperative steroids may decrease the inflammation effect and the release of pro-inflammatory mediators like IL-1 and TNF that accompany the surgical process. [2-5] These effects may shorten the ICU-LOS and the mechanical ventilation period. In addition, table 2 presented higher ICU-LOS and mechanical ventilation period in the control group. Partial explanation could be the lack of systemic steroids in this group. Therefore, we believe that inhaled steroids can not give the entire anti-inflammatory effect that systemic steroids achieved.

Our study has some limitations. First, the small number of patients may have led to a lower statistical power of the variables. Second, the patients were followed for only 2 weeks after hospital discharge. Nevertheless, preoperative steroids proved to be an important adjunct

In conclusion, patients with COPD are at higher risk for post-CABG complications, particularly nonpulmonary ones, such as atrial tachyarrhythmias and wound infection. The administration of preoperative systemic steroids may be of benefit in reducing their postoperative ICU and hospital stay. Larger-scale studies that include more patients with severe COPD are needed to confirm our results.

## Competing interests

The author(s) declare that they have no competing interests.

## Authors' contributions

Daniele Starobin: Design, aquired data, design, drafted paper

David Shitrit: drafted paper, analyzed data

Moshe Garty: Conceived, interpretated data

Mordechai R Kramer: Approval of final version, conceived

## References

[B1] Smetana GW (1999). Preoperative pulmonary evaluation. N Engl J Med.

[B2] Kroenke K, Lawrence VA, Theroux JF, Tuley MR (1992). Operative risk in patients with severe obstructive pulmonary disease. Arch Intern Med.

[B3] Pien LC, Grammer LC, Patterson R (1988). Minimal complications in a surgical population with severe asthma receiving prophylactic corticosteroids. J Allergy Clin Immunol.

[B4] Kabalin CS, Yarnold PL, Grammer LC (1995). Low complication rate of corticosteroid-treated asthmatics undergoing surgical procedures. Arch Intern Med.

[B5] Paone G, Higgins RSD, Havstad SL, Silberman AN (1998). Does age limit the effectiveness of clinical pathways after coronary artery bypass graft surgery?. Circulation.

[B6] Tarhan S, Moffit EA, Sessler AD, Douglas WW, Taylor WF (1973). Risk of anesthesia and surgery in patients with chronic bronchitis and chronic obstructive pulmonary disease. Surgery.

[B7] Cain HD, Stevens PM, Adaniya R (1979). Preoperative pulmonary function and complications after cardiovascular surgery. Chest.

[B8] Jackson MCV (1988). Preoperative pulmonary evaluation. Arch Intern Med.

[B9] Oh SH, Patterson R (1974). Surgery in corticosteroid-dependent asthmatics. J Allergy Clin Immunol.

[B10] (1987). Standards for the Diagnosis and Care of Patients with Chronic Obstructive Pulmonary Disease (COPD) and Asthma. Am Rev Resp Dis.

[B11] Kurki TSO, Kataya M (1996). Preoperative prediction of postoperative morbidity in coronary artery bypass grafting. Ann Thorac Surg.

[B12] Hotchkiss RS (1988). Perioperative management of patient with chronic obstructive pulmonary disease. Int Anesth Clin.

[B13] Epstein SK, Faling J, Daly BDT, Celli BR (1993). Predicting complications after pulmonary resection. Chest.

[B14] Gracey DR, Divertie MB, Didier EP (1979). Preoperative pulmonary preparation of patients with chronic obstructive pulmonary disease. Chest.

[B15] Magovern JA, Sakert T, Magovern GJ (1996). A model that predicts morbidity and mortality after coronary artery bypass graft surgery. J Am Coll Cardiol.

[B16] Hallett JW, Bower TC, Cherry KJ, Gloviczki P, Joyce JW, pairolero PC (1994). Selection and preparation of high-risk patients for repair of abdominal aortic aneurysms. Mayo Clin Proc.

[B17] Sauerland S, Nagelschmidt M, Mallmann P, Neugebauer EA (2000). Risks and benefits of preoperative high dose methylprednisolone in surgical patients: a systematic review. Drug Saf.

